# An Assessment of GPT-3.5 and GPT-4.0 Responses to Scoliosis FAQs

**DOI:** 10.3390/jpm16040206

**Published:** 2026-04-07

**Authors:** Tu-Lan Vu-Han, Enikö Regényi, Vikram Sunkara, Paul Köhli, Friederike Schömig, Alexander P. Hughes, Michael Putzier, Matthias Pumberger, Thilo Khakzad

**Affiliations:** 1 Center for Musculoskeletal Surgery (CMSC), Campus Mitte, Charité—Universitätsmedizin Berlin, Charitéplatz 1, 13353 Berlin, Germany; 2 Berlin Institute of Health at Charité—Universitätsmedizin Berlin, BIH Biomedical Innovation Academy, Charitéplatz 1, 10117 Berlin, Germany; 3 Explainable AI for Biology, Zuse Institute Berlin, Takustraße 7, 14195 Berlin, Germany; 4 Department of Orthopaedic Spine Surgery, Hospital for Special Surgery, Weill Cornell Medicine, New York, NY 10021, USA

**Keywords:** large language models, chatbots, human and machine interactions, health care

## Abstract

**Background**: ChatGPT is a large language model (LLM) online chatbot developed by OpenAI and launched in November 2022. Early adoption studies have shown high readiness to use this technology for health-related questions and self-diagnosis. However, the quality and clinical adequacy of health-related responses remain incompletely characterized. This study aimed to explore responses generated by ChatGPT-3.5 and ChatGPT-4.0 to common patient questions regarding scoliosis. **Methods**: Ten scoliosis-related frequently asked questions (FAQs) were selected from a larger pool of over 250 patient-facing questions compiled from 17 publicly available FAQ webpages and informed by a Google Trends analysis. Questions were harmonized, grouped by theme, and then reduced by rule-based expert review to a final set intended to represent common patient concerns. **Results**: The median ratings of ChatGPT-3.5 and ChatGPT-4.0 responses ranged from satisfactory, requiring minimal (2) to moderate clarification (3). Across the ten matched questions, no statistically detectable difference was found between models in this study setting (*W* = 8.0, *p* = 0.59; Cliff’s δ = −0.12 [95% CI −0.58, 0.40]); however, given the small question set, unblinded rating process, and poor inter-rater reliability, this should not be interpreted as evidence of equivalence, non-inferiority, or comparable model performance. The results apply only to the 10–15 April 2024, online snapshots of ChatGPT-3.5 and ChatGPT-4.0 and should not be generalized to later model iterations. **Conclusions**: This study should be interpreted as a clinically oriented observational report, intended to inform physician awareness and patient-physician communication rather than validate chatbot accuracy or safety. In this 10–15 April 2024, sample, both model outputs frequently required clinician clarification. Given the small FAQ set, low inter-rater reliability, unblinded design, and single-sample outputs, the findings do not establish equivalence or superiority and apply only to the specific 10–15 April 2024, model snapshots and evaluated questions.

## 1. Introduction

Chat Generative Pre-Trained Transformer (ChatGPT-3.5), commonly known as ChatGPT, is a large language model (LLM) online chatbot by OpenAI. Since its launch in November 2022, the chatbot has seen rapid global adoption, reaching over 100 million users within two months and approximately 1.5 billion visits per month (data current as of September 2023). Early performance analyses reported that ChatGPT can pass common state exams, including medical licensing exams [1,2]. The updates include the implementations of voice and image capabilities, allowing users to interact more intuitively and have a voice conversation. GPT-4.0, the updated version available to paid users, has outperformed medical students and surgery residents on written board-like questions and medical licensing exams [1,2]. Given such performance results, ChatGPT has generated considerable interest regarding its potential applications in health care [3,4], where patients’ informed decision-making is a prerequisite before elective surgery. Around 80% of patients turn to search engines, such as Google, Bing, Ask.com, Yahoo!, and DuckDuckGo, as well as social media to attain knowledge and understanding of their conditions [5,6]. Patients are now turning to chatbots like ChatGPT, given their highly interactive nature. An early cross-sectional survey study performed on 607 individuals found that 78.4% were willing to use ChatGPT for self-diagnosis [7], demonstrating readiness to consult generative artificial intelligence (AI) systems regarding health-related questions. However, prior studies have shown mixed results: it may also produce incomplete or misleading statements, a phenomenon often referred to as ‘hallucination’. Previous studies have demonstrated that chatbots may excel in some fields, while failing to achieve a passing grade in others [8]. In addition, assessments of ChatGPT applications in health care have been both promising and concerning [9,10,11,12], some authors noting the issues of hallucinations and misinformation [13,14]. Studies that evaluate the quality of ChatGPT responses and interactions are needed to explore areas for implementation and limitations of the technology in orthopedic surgery. This exploratory study aimed to describe how two 10–15 April 2024, ChatGPT model snapshots responded to common scoliosis-related patient questions and to assess the perceived adequacy of those responses as judged by expert spine surgeons. The purpose was not to validate chatbot accuracy or safety, not to establish comparative model performance, but rather to provide a time-limited descriptive snapshot of information that patients may encounter and bring into clinical discussions. Because patients already use LLM-based tools to seek health information regardless of physician endorsement, understanding the content and limitations of such responses is clinically relevant to spine care professionals who must contextualize AI-generated information during patient encounters.

## 2. Material and Methods

### 2.1. Mapping FAQs on the Topic ‘Scoliosis’

We reviewed publicly available scoliosis FAQ pages from 17 websites of medical centers and spine programs, yielding a pool of more than 250 patient-facing questions. Source webpages are listed in Supplementary S1. Extracted questions were compiled, deduplicated, and harmonized for overlapping wording. Questions were then grouped into recurring thematic categories identified through manual review informed by word-frequency analysis and Google Trends. From these categories, ten questions were selected by expert review to represent common patient concerns across diagnosis, etiology, progression, nonoperative management, surgery, symptoms, and postoperative recovery. The final selected questions are presented in Supplementary Tables S2 and S3, respectively. This process was intended to create a concise, theme-representative question set rather than an exhaustive or formally validated questionnaire. Because no Delphi process or external validation framework was used, the final FAQ set should be understood as a pragmatic, expert-curated sample rather than a definitive representation of all scoliosis-related patient questions.

### 2.2. Generating Prompts and ChatGPT Responses

The selected FAQs were posed to two different versions of the chatbot without prior prompts or follow-up prompts. The analyses used the public ChatGPT-3.5 and ChatGPT-4.0 web interfaces and the model options available through that interface during the data-collection window of 10–15 April 2024. The models identified in the interface at that time were GPT-3.5 (default) and GPT-4.0 (ChatGPT Plus, interface-labeled April 2024 update). These models were selected because they were the most accessible and widely used generative AI systems at the time. Later model iterations were not evaluated because the study was intentionally designed as a snapshot of responses generated during this predefined collection period. Each of the ten scoliosis-related questions were entered exactly once per model, without prior context or additional prompting to emulate a typical patient inquiry. Only one response per question per model was collected. Because LLM outputs are nondeterministic and may vary across sessions, repeated sampling would be required to estimate typical response behavior; therefore, the present analysis reflects single-sample outputs rather than the full distribution of possible answers. The exact wording of each prompt is shown in Table 1, Table 2, Table 3, Table 4, Table 5, Table 6, Table 7, Table 8, Table 9 and Table 10 and Tables S2 and S3. Because outputs from ChatGPT are nondeterministic and may vary across sessions or model snapshots, we treated this limitation analytically and discuss it explicitly below. The responses were collected, and the questions and corresponding answers were presented to a panel of six orthopedic surgeons, specialized in spine surgery.

### 2.3. Expert Reviews of ChatGPT Responses

Six independent spine surgeons (three attending and three senior resident surgeons) rated each chatbot-generated answer on a 4-point Likert scale using a structured electronic form. Ratings were provided individually, without discussion or consensus. No reconciliation or averaging was performed beyond the statistical aggregation described below. A score of 1 corresponds with ‘excellent response not requiring clarification’, 2 corresponding with ‘satisfactory requiring minimal clarification’, 3 corresponding with ‘satisfactory requiring moderate clarification’, and 4 corresponding with ‘unsatisfactory requiring substantial clarification’. Raters were not involved in the authorship or generation of chatbot responses and provided their assessments independently without discussion or consensus scoring. Raters were not blinded to the model version (ChatGPT-3.5 vs. GPT-4.0). Consequently, expectation bias may have influenced ratings in either direction. No formal rater-calibration session was performed before scoring. To enhance reproducibility, the rating rubric used during assessment is now included in Supplementary S4. The rubric was intended to capture perceived clinical adequacy of patient-facing responses, but it does not constitute a validated psychometric instrument.

### 2.4. Statistical Analysis

Because each question had matched ChatGPT-3.5 and GPT-4.0 answers, we compared per-question median ratings using a Wilcoxon signed-rank test. Inter-rater reliability was assessed using a two-way random-effects, absolute-agreement, single-measure intraclass correlation coefficient (ICC (2,1)) with 95% confidence intervals obtained via nonparametric bootstrap resampling (B = 3000). Given the low ICC, all comparative interpretations were treated cautiously, and the study was not interpreted as supporting equivalence or superiority between models. As a sensitivity analysis, a mixed-effects model was fit with rating as the dependent variable, model version as a fixed effect, and random intercepts for rater and question. As a nonparametric measure of effect size, Cliff’s delta (δ) was calculated for paired median ratings, with 95% confidence intervals obtained by bootstrap resampling (5000 iterations). Values of |δ| < 0.147, 0.33, and 0.474 were interpreted as negligible, small, and medium effects, respectively. Statistical analyses were performed in Python 3.11.0 (pandas 2.2.1, NumPy 1.26.4, SciPy 1.12.0, statsmodels 0.12.2) and figures were created using matplotlib 3.8.4 and seaborn 0.13.2 [15].

### 2.5. In-Depth Analysis of ChatGPT Responses

To contextualize the chatbot responses, the authors qualitatively compared each answer with contemporary concepts in scoliosis diagnosis and management as reflected in the cited literature. This component was intended as a narrative clinical interpretation and not as a formal guideline-concordance audit.

## 3. Results

We compiled the scoliosis FAQs from over seventeen different websites of spine specialists in a list of over 250 questions. From these questions, we derived common themes that we quantified by generating a word cloud text analysis. Finally, we selected a total of ten questions from the FAQs to address a range of common scoliosis questions.

### 3.1. Response Ratings

**Figure 1:**
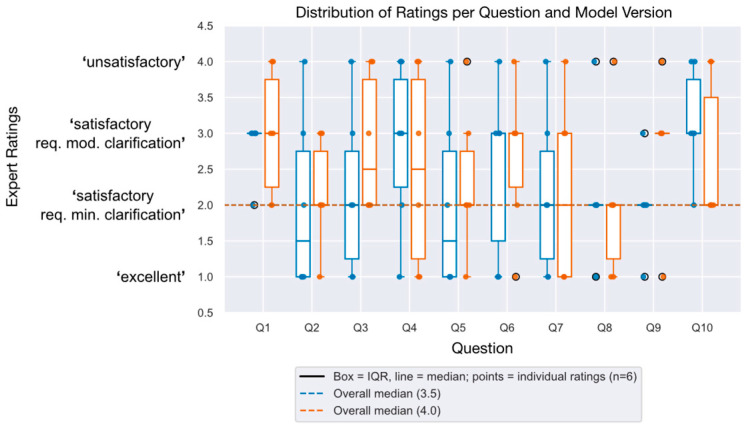
Distribution of surgeon ratings for each question and model version. In each boxplot, the box indicates the interquartile range (IQR). The horizontal line within the box represents the median, and the whiskers extend to the minimum and maximum observed ratings. Points denote individual surgeon ratings (*n* = 6) for each question. Dashed horizontal lines indicate the overall median rating for ChatGPT-3.5 (blue) and GPT-4.0 (orange). Ratings were assigned by unblinded surgeon raters using a 4-point adequacy rubric (1, ‘excellent response not requiring clarification’ to 4, ‘unsatisfactory, requiring substantial clarification’).

Across all ten matched scoliosis questions, median ratings for both models did not differ significantly (Wilcoxon signed-rank: *W* = 8.0, *p* = 0.589). Inter-rater reliability was poor (ICC (2,1)) = 0.04, 95% CI [−0.02, 0.10]; 20 targets rated by 6 raters. In the mixed-effects sensitivity analysis, model version was not a significant predictor of rating (β = 0.13 ± 0.11, *p* = 0.20). The effect size analysis showed a negligible difference between models (Cliff’s delta = −0.120, 95% CI [−0.580, 0.400]). Given the small number of matched questions (*n* = 10), the study was underpowered to detect small effects (power < 0.4 for medium effect size). These findings indicate that no statistically detectable difference was observed in this dataset; however, the small FAQ sample, poor inter-rater reliability, and unblinded ratings preclude any conclusion regarding equivalence, superiority, or reliably comparable model performance. The low ICC can be interpreted in two ways: first, as evidence that the rubric and rating construct allowed substantial variation in interpretation; and second, as a clinically relevant finding that expert surgeons may differ in what they consider an adequate patient-facing explanation (Figure 1).

Here we present each question and response (Q-R) along with our in-depth analysis of the response quality. The asterisk (*) marks responses that included a referral to a health professional or spine specialist.

**Table 1:** Responses to Question 1—What is Scoliosis?

*Question 1:*	*What Is Scoliosis?*
GPT-3.5 response:	“Scoliosis is a medical condition characterized by an abnormal lateral (sideways) curvature of the spine. This curvature can be in the shape of an “S” or a “C” and may develop in various degrees of severity. It often occurs during growth spurts in childhood or adolescence but can also result from other factors, and it can affect individuals of all ages.”
GPT-4.0 response:	“Scoliosis is a medical condition where there is an abnormal lateral (sideways) curvature of the spine. When viewed from the back, a typical spine is straight. In a person with scoliosis, the spine can curve to the left, right, or both, often resembling an “S” or “C” shape.”

#### 3.1.1. Analysis of Q1-R1

Both chatbots, GPT-3.5 and GPT-4.0, mention the abnormal curvature of the spine on an anteroposterior radiograph. However, both fail to mention that scoliosis is a three-dimensional condition [16,17]. The differentiation between structural and non-structural curves is essential for scoliosis classification and subsequent treatment strategies [18,19]. These answers would require clarification and guidance by a medical professional. ChatGPT-3.5 mentions that different factors can lead to scoliosis. The median ratings of ChatGPT-3.5 and ChatGPT-4.0 answers to this question were 3.0 for both models, corresponding with ‘satisfactory requiring moderate clarification’.

**Table 2:** Responses to Question 2—How Can I Prevent Scoliosis? The asterisk (*) marks responses that included a referral to a health professional or spine specialist.

*Question 2:*	*How Can I Prevent Scoliosis?*
GPT-3.5 response *:	“Scoliosis is primarily a structural issue of the spine, and there are no known ways to prevent it through lifestyle choices or behaviors. Some cases of scoliosis have genetic or hereditary components, making prevention impossible. Regular medical check-ups and early detection, however, can help manage the condition more effectively if it does develop.”
GPT-4.0 response *:	“Many cases of scoliosis are idiopathic, meaning the cause is unknown, so prevention is not always possible. However, early detection and appropriate management can prevent progression in some cases. Regular check-ups during growth spurts in children can be helpful.”

#### 3.1.2. Analysis of Q2-R2

The chatbots explain that the cause of scoliosis is often unknown, thus referring to ‘idiopathic scoliosis’, of which adolescent idiopathic scoliosis makes up 85% of scoliosis types [20] and has an incidence of 2.5–4.5% [17]. Both chatbots emphasize that early scoliosis detection and management are key [21]. ChatGPT-3.5 mentions that the structural issue of the spine is a primary characteristic [16]. These answers received a median rating of 2.0 by the spine specialists.

**Table 3:** Responses to Question 3—Can Scoliotic Curves Progress? The asterisk (*) marks responses that included a referral to a health professional or spine specialist.

*Question 3:*	*Can Scoliotic Curves Progress?*
GPT-3.5 response *:	“Yes, scoliotic curves can progress, especially during periods of rapid growth, such as adolescence. The degree of progression varies from person to person and depends on factors like the type of scoliosis, the age of onset, and treatment interventions. Regular monitoring by a healthcare professional is essential to track curve progression.”
GPT-4.0 response *:	“Yes, scoliotic curves can progress, especially during rapid growth periods in adolescence. Regular monitoring by a healthcare professional is important to determine the progression and decide on appropriate treatment.”

#### 3.1.3. Analysis of Q3-R3

Both chatbot responses described that scoliotic curves can progress and emphasize the role of rapid growth periods in adolescence [22]. Strong predictors of curve progression include magnitude of the curve and age at presentation, skeletal maturity [23]. ChatGPT-3.5 lists several factors that can accelerate curve progression, including the type of scoliosis, age of onset, and ongoing treatment interventions. The reviewers rated the responses with a median score of 2.0 and 2.8, respectively.

**Table 4:** Responses to Question 4—Do I need Scoliosis Surgery? The asterisk (*) marks responses that included a referral to a health professional or spine specialist.

*Question 4:*	*Do I Need Scoliosis Surgery?*
GPT-3.5 response:	“Whether or not you need scoliosis surgery depends on several factors, including the severity of your spinal curvature, your age, and whether non-surgical interventions have been effective in managing the condition. Surgical intervention is usually considered when the curvature becomes severe (typically over 40–50 degrees) and is accompanied by other factors like pain, breathing difficulties, or functional limitations.”
GPT-4.0 response *:	“The decision for scoliosis surgery is based on several factors, including the severity and location of the curve, the patient’s age, and the risk of progression. Generally, surgery is considered for curves that are greater than 40–50 degrees or if the curve is progressing despite other treatments. An orthopedic surgeon specializing in spine disorders can provide guidance.”

#### 3.1.4. Analysis of Q4-R4

Here, ChatGPT-3.5 and ChatGPT-4.0 list relevant factors that may indicate scoliosis surgery. In line with the current literature, Cobb angles over 40–50 degrees are often considered for scoliosis surgery in idiopathic scoliosis [22]. Pain and breathing difficulties may relate to more severe thoracic curves, such as Cobb angles above 80° [24]. However, the chatbots do not differentiate between different scoliosis types, which inform the spectrum of available treatment strategies. Here, ChatGPT-4.0 refers to the orthopedic surgeon who specializes in spine disorders. The reviewers rated the responses to this question with a median of 3.0 and 2.5, respectively, corresponding with ‘satisfactory, requiring moderate clarification’.

**Table 5:** Responses to Question 5—How Can I Avoid Scoliosis Surgery? The asterisk (*) marks responses that included a referral to a health professional or spine specialist.

*Question 5:*	*How Can I Avoid Scoliosis Surgery?*
GPT-3.5 response *:	“Avoiding scoliosis surgery may be possible through non-surgical interventions if the curvature is detected early and is not too severe. These interventions may include bracing, physical therapy, and exercises to help stabilize or reduce the curve. Regular follow-up with a spine specialist is essential to monitor progress and adjust the treatment plan as needed.”
GPT-4.0 response *:	“Non-surgical treatments, like bracing or physical therapy, can sometimes help manage scoliosis and prevent its progression, particularly in mild to moderate cases. Adherence to prescribed treatments and regular follow-ups are crucial.”

#### 3.1.5. Analysis of Q5-R5

Here, both chatbots state that scoliosis progression may be prevented by non-surgical treatment strategies. In addition, ChatGPT-3.5 states that a timely detection and curve severity may play a role in predicting the outcome of non-surgical treatments [25]. Indeed, younger idiopathic scoliosis patients with low skeletal maturity and lower Cobb angles are most likely to respond to non-surgical therapy [26]. While only around 0.23% of adolescent idiopathic scoliosis require treatment [27], non-surgical interventions in neuromuscular scoliosis have limited effects and function as interim solutions until surgical therapy can occur [28]. Both chatbots recommend adhering to the treatment plan and regular visits to the spine specialist, required for effective treatment. The reviewers rated the chatbot responses with a median score of 2.0 and 2.0, respectively, corresponding with ‘satisfactory, requiring minimal clarification’.

**Table 6:** Question 6—What Sports Can I do With Scoliosis? The asterisk (*) marks responses that included a referral to a health professional or spine specialist.

*Question 6:*	*What Sports Can I do With Scoliosis?*
GPT-3.5 response *:	“Many individuals with scoliosis can participate in sports and physical activities. The choice of sports will depend on the severity of the scoliosis, the level of pain or discomfort, and the advice of your healthcare provider. Low-impact sports like swimming and yoga are often recommended, but it’s essential to consult with a healthcare professional for personalized guidance.”
GPT-4.0 response *:	“Many people with scoliosis can participate in a wide range of sports. The type and severity of scoliosis, as well as the specific sport in question, will influence this. It’s essential to consult with a healthcare provider to determine which activities are safe and beneficial.”

#### 3.1.6. Analysis of Q6-R6

The current literature and guidelines for patients with idiopathic scoliosis clearly recommend physical activity [29,30], some guided exercises having the potential to reduce curve progression [31] or alleviate associated lower back pain [32]. However, there is a scarcity of high-quality evidence regarding the specific recommended physical activities [30], with some authors recommending more restrictive activities and avoidance of high-impact sports for patients with more severe curves [33]. Here, both chatbots refer to a healthcare professional. The responses received median ratings of 3.0 and 3.0, respectively, requiring moderate clarification.

**Table 7:** Responses to Question 7—What Causes Scoliosis? The asterisk (*) marks responses that included a referral to a health professional or spine specialist.

*Question 7:*	*What Causes Scoliosis?*
GPT-3.5 response *:	“The exact causes of scoliosis can vary. Some cases are idiopathic, meaning the cause is unknown. Other potential causes include genetics, neuromuscular conditions, congenital spine abnormalities, or traumatic injuries. Understanding the cause of scoliosis in a specific individual often requires a thorough evaluation by a healthcare provider.”
GPT-4.0 response:	“The exact cause of scoliosis is unknown in most cases and is termed “idiopathic.” However, scoliosis can be associated with conditions like cerebral palsy, muscular dystrophy, genetic conditions, or birth defects. Some cases may also result from injuries, infections, or tumors.”

#### 3.1.7. Analysis of Q7-R7

Both chatbots list different causes for scoliosis, emphasizing that the causes are mostly unknown—in line with the current literature [34]. ChatGPT-4.0 states that most of the scoliosis cases are ‘idiopathic’, in line with the literature [34]. Other causes, listed by both chatbots, include genetic causes [20], neuromuscular conditions [35], such as cerebral palsy, and muscular dystrophy, congenital spine abnormalities, birth defects, traumatic injuries infections and tumors [36]. The lists are incomplete in both chatbots. ChatGPT-3.5 refers to a healthcare provider for individual evaluation and diagnosis. Interestingly, ChatGPT-4.0 does not. The answers were rated with a median of 2.0 and 2.2, respectively, corresponding with ‘satisfactory, requiring minimal clarification’.

**Table 8:** Question 8—What Are Common Symptoms of Scoliosis?

*Question 8:*	*What Are Common Symptoms of Scoliosis?*
GPT-3.5 response:	“Common symptoms of scoliosis may include an uneven shoulder or hip height, an asymmetrical appearance of the back when bending forward, and in some cases, back pain, or discomfort. However, scoliosis is not always accompanied by noticeable symptoms, especially in mild cases.”
GPT-4.0 response:	“Symptoms can vary based on the severity, but common signs include an uneven waist, leaning to one side, asymmetry in shoulder or hip height, and uneven shoulder blades. Some people may experience back pain or respiratory issues in severe cases.”

#### 3.1.8. Analysis of Q8-R8

Both chatbots list clinical symptoms associated with scoliosis. ChatGPT-4.0 points out that symptoms may vary based on severity and mentions back pain and respiratory issues. Here, neither model refer to a medical professional for evaluation. The reviewers rated both responses with a median of 2.0.

**Table 9:** Responses to Question 9—How Dangerous is Scoliosis Surgery? The asterisk (*) marks responses that included a referral to a health professional or spine specialist.

*Question 9:*	*How Dangerous Is Scoliosis Surgery?*
GPT-3.5 response *:	“Scoliosis surgery is generally considered safe when performed by experienced surgeons in appropriate candidates. Like any surgical procedure, there are risks, including infection, bleeding, complications with anesthesia, and, in rare cases, neurological or vascular complications. The decision to undergo surgery should be made after a thorough evaluation and discussion of potential risks and benefits with a healthcare provider.”
GPT-4.0 response:	“All surgeries carry risks. Common risks with scoliosis surgery include bleeding, infection, nerve damage, and problems with the hardware used to correct the spine. Modern surgical techniques have made the procedure safer, but the outcome can vary based on the individual’s health, the severity of the scoliosis, and the surgeon’s experience.”

#### 3.1.9. Analysis of Q9-R9

The safety of scoliosis surgery has significantly improved in the past decades [37], however, it may vary, depending on the individual, surgical indication, and extent of surgery, i.e., levels of spinal fusion [38,39,40]. A survey by the Scoliosis Research Society found relatively low surgical risks with neurological complications in 0.72% of surgically treated patients [41]. In the response, ChatGPT-3.5 refers to a medical professional, whereas ChatGPT-4.0 mentions that outcomes may vary based on the individual’s health. The median response ratings were 2.0 and 3.0, respectively.

**Table 10:** Responses to Question 10—What Movements Will I Be Able to Do After Scoliosis Surgery?

*Question 10:*	*What Movements Will I Be Able to Do After Scoliosis Surgery?*
GPT-3.5 response:	“The ability to move after scoliosis surgery depends on the surgical technique used, the location and extent of the spinal fusion, and individual.”
GPT-4.0 response:	“Post-surgical mobility varies depending on the type of surgery and the individual’s recovery. Initially, movements might be limited to ensure proper healing. Over time, and with physical therapy, many people regain significant mobility and can engage in everyday activities. Some high-impact activities or extreme spinal motions might be restricted permanently or for an extended period after surgery.”

#### 3.1.10. Analysis of Q10-R10

Both responses to this question require further specification. In the authors’ clinical interpretation, these responses would likely require clarification during patient counseling. The question aimed at returning to physical activity and sports after spinal fusion, a subject that currently lacks standard guidelines and a substantial body of evidence [42]. However, the current consensus is that physical activities are generally recommended, and specific exercises should be performed under the guidance of a spine surgeon [43]. Some studies have associated early rehabilitation with improved postoperative outcomes [44]. In addition, return to sports is rather a matter of time and level of impact [45,46,47]. The ChatGPT-3.5 response may suggest limited mobility after spinal fusion overall, which may be misleading or incomplete considering the cited literature. ChatGPT-4.0 points out that initial mobility may be limited, but there may be improvement over time with proper healing. The reviewers rated the responses with 3.0 and 2.0.

## 4. Discussion

The study should be interpreted as a descriptive, clinically oriented observational report rather than a validation study of chatbot accuracy, safety, or comparative performance. Its purpose was to characterize the kinds of scoliosis-related responses a patient might have encountered from two ChatGPT model snapshots during April 2024 and to examine how these responses were perceived by spine surgeons. In this narrow context, the answers were often understandable but frequently incomplete and commonly required clinician clarification. The clinical relevance of this work lies in helping physicians anticipate the AI-generated information that patients may bring into consultations [48].

At the same time, the methodological limitations materially restrict the strength of any inference. The low inter-rater reliability indicates substantial disagreement in the application of the rating construct, and the unblinded design leaves the results vulnerable to expectation bias. The FAQ set was narrow and expert-curated rather than formally validated, and only a single output per question per model was assessed despite the nondeterministic behavior of LLMs. For these reasons, the study should not be interpreted as establishing reliable comparative performance between models.

The diagnosis of scoliosis and subsequent treatment can be devastating to some patients, as the condition often entails long-term treatment, regular visits to a health practitioner to monitor curve progression, with the potential outcome of surgical treatment. Scoliosis treatment strategies remain complex and depend on the age and time of diagnosis, as well as scoliosis (sub-)type. The chatbots did not differentiate between the scoliosis subtypes and remained general and vague in their responses, often referring to a health care professional. As such, the explored model versions could not be relied on for nuanced clinical guidance. These observations underscore the continued importance of physician oversight when patients interpret online or AI-generated information.

Informed consent before elective surgery is an important pillar in clinical practice that strengthens patients’ autonomy in preparation for treatment [49]. Previous studies have shown that orally delivered pre-operative information served patients better than written and read information [50]. How the capabilities of chatbots may alter patient–physician interactions has yet to be explored extensively. Currently, the dissemination of adequate information on the nature, duration, pros and cons, post-operative state, and alternatives of the planned procedure remains with the operating surgeon [51]. This variability was reflected in the spread of surgeon ratings. Importantly, that variability may indicate both a measurement problem and a clinically meaningful phenomenon: on one hand, the rubric may not have constrained judgements tightly enough to yield consistent scoring; on the other hand, different surgeons may genuinely hold different expectations regarding what constitutes an adequate patient-facing explanation for informed decision-making. The low ICC therefore limits the study’s comparative interpretability while also highlighting heterogeneity in expert expectations. In line with this notion are other studies that have found ChatGPT performs poorly on questions related to informed consent [52], undermining that guidance by a medical professional is still needed.

The overall readiness of ChatGPT users to consult the chatbots regarding health-related questions is high (78.4%) [7]. In turn, the distribution of misinformation and poor performance may lead to the erosion of user trust [53]. At this point in time, our understanding of ChatGPT behavior in specific fields is yet underexplored. We observed the inherent randomness of ChatGPT, which causes the generation of different responses to repeat questions. The publicly available models (GPT-3.5 and GPT-4.0) both suffer from limitations such as “hallucinations” and do not learn from experience. It is advised to take caution, especially in fields where answers need to be accurate and exhaustive [54]. In addition, OpenAI is constantly improving its technology, resulting in a newer version of ChatGPT that gives more extensive replies, of which the accuracy of content and subsequent interpretation by humans of different educational backgrounds has yet to be explored. Differences in phrasing style, depth of explanation, and tone across chatbot answers may have contributed to divergent perceptions of adequacy. Incomplete or imprecise explanations in chatbot outputs may influence patients’ perception of disease severity, diagnostics, and treatment options. Anchoring chatbot-based patient education to current evidence and established guidelines remains essential.

## 5. Limitations

This study has several limitations that materially restrict interpretation. First, the evaluation was based on a small, expert-curated set of ten scoliosis-related FAQs derived from a larger pool of publicly available questions. Although the selection process was structured and reported transparently, the final set was not developed through a Delphi process or formal validation framework and therefore represents a pragmatic sample rather than a comprehensive patient-question instrument. Second, only one response per question per model was collected. Because LLM outputs are nondeterministic and may vary across sessions and model updates, these single-sample outputs cannot be assumed to represent typical or stable model behavior. Repeated sampling would be required to characterize response variability and central tendency. Third, raters were not blinded to model identity, which introduces the possibility of expectation bias in either direction. In addition, no formal rater-calibration process was performed before scoring. Fourth, inter-rater reliability was poor (ICC (2,1) = 0.04), indicating substantial disagreement among raters. This low agreement may reflect both limitations of the rubric as a measurement construct and real differences among surgeons in their expectations for adequate patient-facing explanations. Regardless of cause, the low ICC substantially limits comparative inference. Fifth, the study assessed perceived answer adequacy rather than factual accuracy, clinical safety, patient comprehension, or downstream patient outcomes. Accordingly, the manuscript should be interpreted as a descriptive, time-limited benchmark rather than as a validation of chatbot performance. Future work should incorporate blinded raters, rater calibration, repeated output sampling, larger and more diverse question sets, and more rigorous psychometric or hierarchical modeling approaches.

## 6. Conclusions

This study provides a time-limited descriptive snapshot of how two ChatGPT model versions on 10–15 April 2024 responded to a small set of common scoliosis-related patient questions. Under the present study conditions, no statistically detectable difference was observed in surgeon ratings between the two model snapshots; however, this should not be interpreted as evidence of equivalence, superiority, or comparable performance. The findings of this study are constrained by the small FAQ set, single-sample outputs, an unblinded ratings process, and poor inter-rater reliability. The principal contribution of the study is clinical rather than comparative: it highlights the kinds of incomplete, generalized, or potentially misleading information that patients may encounter when consulting LLM-based tools about scoliosis. It underscores the continuing need for physician interpretation and individualized counseling. The findings apply only to the 10–15 April 2024, online model snapshots and the specific FAQ set evaluated here.

## Data Availability

The original contributions presented in this study are included in the article/Supplementary Material. Further inquiries can be directed to the corresponding authors.

## Supplementary Materials

The following supporting information can be downloaded at: https://www.mdpi.com/article/10.3390/jpm16040206/s1, S1: List of All Scoliosis FAQ; Table S2: ChatGPT-3.5 Responses; Table S3: ChatGPT-4.0 Responses; S4: Rating Rubric.

## Abbreviations

AI	artificial intelligence
ChatGPT	Chat Generative Pre-Trained Transformer
FAQ	frequently asked questions
ICC	intraclass correlation coefficient
LLM	large language model
