# Supplementary material for: An Assessment of GPT-3.5 and GPT-4.0 Responses to Scoliosis FAQs
Source: J Pers Med. 2026 Apr 7;16(4):206. doi: 10.3390/jpm16040206 (PMC13117403; doi:10.3390/jpm16040206)
Supplement: Supplementary file 1 [file jpm-16-00206-s001.zip › S1_all_scoliosis_FAQs.pdf]

Supplement 1: Scoliosis FAQs

1. https://orthoinfo.aaos.org/en/diseases--conditions/scoliosis-frequently-asked-questions

What is scoliosis?
What is the difference between idiopathic scoliosis and other types of scoliosis?
How serious is adolescent idiopathic scoliosis?
Does scoliosis cause back pain?
Can scoliosis curves get better on their own?
What can i do to prevent my child's scoliosis from getting worse?
Is it safe for my child to exercise and participate in sports?
Will my child be able to live a normal life?
Does scoliosis run in families?
If i have scoliosis, will my children have it?
Does my child's bad posture cause the scoliosis?
Does a leg length difference cause or worsen the curve?
Do sports activities or heavy backpacks cause scoliosis?
Is scoliosis related to an injury?
Could i have prevented my scoliosis?
How early should children be screened for scoliosis?
Do siblings of children with scoliosis need to be checked?
When should the child of parents with scoliosis be examined?
What health problems might i have later in life as a result of scoliosis?
Will i have a hump on my back when i get older?
One of my hips looks higher than the other. Can anything correct this?
Will having scoliosis affect my ability to bear and deliver a child?
Can i have an epidural in the future?
Does having scoliosis make me more prone to osteoporosis?
Will having scoliosis influence what i do later in life, such as what jobs i can do?
Will the metal detectors go off in airport security after i have rods placed in my spine?
What are treatment options for scoliosis?
What will make the spine go back to being straight again?
What happens if the child has just a slight curve?
Will having so many x-rays cause future health problems?
What happens if no treatment is done? Will the curve get worse?
Do i have to wear a brace if i have scoliosis?
For what degree curve do you decide to prescribe a brace?
Does bracing work?
What kind of a brace will i need and why?
What does the brace look like?
How long will i need to wear the brace?
Can i wear it at night only?
What will it be like to wear a brace?

What happens if i don't wear the brace?
Will physical therapy help my scoliosis?
Will chiropractic treatment help?
Will acupuncture help treat and manage my back problems?
Will yoga help treat and manage my back problems?
How can massage help my back problems?
Will pilates make my scoliosis or back problems better?
Do i need scoliosis surgery?
How successful is surgery for scoliosis?
How straight will my spine be after surgery?
I have back pain associated with scoliosis. Will the surgery relieve it?
What is involved with a spinal fusion surgery?
What is a bone graft?
How long does the surgery take?
How much pain will i be in after surgery?
What type of pain control will there be after the operation?
Do the rods and other implants stay in my spine even after it has fused?
Will fusion make my back stiff and unable to move?
Can i have my scoliosis corrected without a fusion?
How long do patients need pain medication after being discharged home?
What limitations will i have right after surgery?
Does surgery lead to permanent restrictions on activities?
Will i be able to walk after surgery?
When can i go back to school after the operation?
When will i be allowed to drive a car?
When can i start hanging out with my friends again?

2. https://ssr.org.uk/scoliosis/faqs/

What is scoliosis?
Why has this happened?
I (or a family member) have been diagnosed with scoliosis. What is going to happen now=
Could i have prevented my scoliosis?
How can i encourage my child to wear their brace?
How long does it take to recover from scoliosis surgery?

3. https://www.ortho.wustl.edu/content/education/3634/patient-education/educational-materials/scoliosis-faqs.aspx

2. What causes some children to develop scoliosis while others do not?
2. What causes some children to develop scoliosis while others do not?
3. What causes adults to develop scoliosis?
4. At what point is a brace needed to treat scoliosis?
5. Beyond bracing, what are the treatment options? Are minimally invasive procedures becoming more common?
6. Can scoliosis cause related problems with blood flow or organ systems?

Why does scoliosis develop most often in late childhood?

4. https://jasonlowensteinmd.com/patient-info/scoliosis-faq/
What is scoliosis?
Is scoliosis genetic?
How should i take care of a child with scoliosis?
What is a cobb angle?
Do scoliotic curves progress?
How important is early-stage scoliosis intervention?
Are there activities that i should avoid if i have scoliosis?
How does sleeping work with scoliosis?
What are conservative treatment options for patients with scoliosis?
What surgical procedures are performed for patients with scoliosis?
How is scoliosis diagnosed?
Is there anything that i should avoid if i have scoliosis?
I have scoliosis. What should i do?
Can scoliosis be caused by my environment?
How much money will spinal fusion surgery cost me?
What are the potential complications with scoliosis surgery?
Do rods break?

5. https://www.scoliosis.org/information/faqs/

Can you please refer me to a doctor for my scoliosis?
My doctor told me not to worry about my scoliosis. What should i do?
Can you tell me what is the best treatment for scoliosis?
I have a mild scoliosis curvature. Should i be concerned?
Will you please send me a description of exercises to help my scoliosis?
Do you think a chiropractor could help my scoliosis?
Will scoliosis affect my ability to have children?
When i was younger my scoliosis didn't bother me, but it has recently become more painful. What should i do?
I have enclosed all the particulars concerning my scoliosis condition. What do you think would be the best treatment for me?
I am preparing a science project on scoliosis and would like to receive information. Can you send me x-rays and braces to illustrate my report?
How can i get in touch others who are dealing with spinal curvatures?
I lost my job and my insurance as a result of my scoliosis. Now i realize treatment is available for adults. Where can i find funds?
Can the national scoliosis help me with my medical expenses?
I have been told that there are college scholarships available for scoliosis patients. Whom do i contact for information?
What is being done to find out what causes idiopathic scoliosis?

6. https://patient.uwhealth.org/education/frequently-asked-questions-about-scoliosis
When is scoliosis first noticed?
When is scoliosis first noticed?

What causes scoliosis?
How is idiopathic scoliosis measured?
What happens to the curves in idiopathic scoliosis?
Why is it important to detect and treat idiopathic scoliosis?
What are the goals of treatment in idiopathic scoliosis?
How is scoliosis treated?

7. https://scoliosisandspinecenter.com/faq/
To whom is bracing applied in scoliosis patients?
Is surgery the only option in scoliosis treatment?
Scoliosis (schroth) exercises:
Kyphosis

8. https://www.childrenshealthireland.ie/list-of-services/scoliosis/frequently-asked-questions-faqs-scoliosis/

Is scoliosis genetic?
Is scoliosis painful?
Is scoliosis dangerous?
Can i exercise and play sport if i have scoliosis?
Are spina bifida and scoliosis related?
What other conditions are linked to scoliosis
What is a cobb angle?

9. https://www.settingscoliosisstraight.org/faq/
What is scoliosis?
How can you tell that i have scoliosis?
What causes scoliosis....ccarrying a heavy backpack, not
Drinking enough milk, poor posture?
Does scoliosis hurt?
How common is scoliosis?
Does scoliosis run in families?
Is there a way to tell if my scoliosis will get worse or not?
Can i still exercise if i have scoliosis?
What is the treatment for scoliosis?
Are there any alternative treatments that may help?
What is the purpose of wearing a brace?
Will a brace make my scoliosis better?
Do i have to wear the brace all of the time?
How long do i have to wear the brace?
What is the goal of surgery?
What kind of surgery will i need?
How long will i be in the hospital if i have surgery?
How much school will i miss if i have surgery?
When can i play sports again?
What will the surgery scar look like?
If 1 have metal in my back, will it set off alarms in the

Airport?
Will | need to have more surgeries later on?
What if i decide not to have surgery?
Are my children going to have scoliosis?
How do i choose a scoliosis surgeon?
10. https://www.hss.edu/health-library/conditions-and-treatments/scoliosis-bracing
What is scoliosis bracing?
How does it work?
What are the different kinds of braces?
How long does it take to fix scoliosis?
What degree of scoliosis requires a brace?
Can I do physical therapy instead?

11. HTTPS://SCOLIOSIS.AE/FAQ/
What is scoliosis?
What are the causes of scoliosis?
Does carrying heavy bag on one shoulder, bad posture of sitting and sleeping cause scoliosis?
How early should children be screened for scoliosis?
How does a doctor diagnose scoliosis?
How will doctor know that child has scoliosis during examination?
How do I know my child's scoliosis is not severe?
Will physical therapy help my scoliosis?
My child has scoliosis, does it mean I need surgery?
What are the long term impact of not treating severe scoliosis?
When will the doctor recommend surgery for scoliosis?
What is the objective of surgery ?
Can I have a normal life after scoliosis corrective surgery?
Does having scoliosis surgery have an effect on pregnancy?

12. HTTPS://EN.ISICO.IT/SCOLIOSIS-FAQ/
What is scoliosis?
How many types of scoliosis are there?
How is scoliosis detected?
Why screen for scoliosis?
Who should be consulted for scoliosis?
What is the forward bend test?
Are x-rays measured too?
What purpose do exercises serve?
Why do some people say exercises are unnecessary?
When is a brace recommended?
How long does it have to be worn each day, on average?
And for how many years?
What happens in the case of adult scoliosis?
Can you do sport if you have scoliosis?
Can you swim if you have scoliosis?

Why don't you use the milwaukee brace?
Can wearing a brace be a problem for teenagers?
When is surgery necessary?
Is the spine put at risk by long periods spent sitting at a desk?
What can schools do to help?
Do “school-induced” orthopaedic problems actually exist?
Are the uni standards, followed by manufacturers, correct?
Can scoliosis be cured?
What results can you expect from treatment for scoliosis?
Is a scoliotic posture/attitude considered a disease?
What do scientific studies say?
Is osteopathy effective in the treatment of scoliosis?
Are all braces the same?
When is surgery inevitable?
What is one of the main complications after scoliosis surgery?
Can you bathe (in the sea or in a swimming pool) with a brace on?
Can you ride a bike, motorbike or moped with a brace on?
Why do the hours of brace wearing have to be continuous?
Are x-rays harmful?
Can a back become bent again once the treatment is finished?
When should a brace be recommended?
Why was plaster casting replaced by the sforzesco brace?
The right way to wear your brace
Can you bathe (in the sea or in a swimming pool) with a brace on?
Do school backpacks cause scoliosis?
Is natural childbirth contraindicated in a woman who has had scoliosis surgery?
Should the siblings of scoliosis patients be screened for the condition?
How should | carry my brace on a plane?
Does scoliosis worsen in menopause?
Scoliosis: can | face multiple pregnancies?
With each new brace, should patients expect the same initial discomfort they experienced with their first one?
A comparison of the chéneau and sforzesco braces
Are postural collapse and vertebral collapse the same thing?
Is anormal back perfectly straight?
What is the real goal of scoliosis treatment?
Physical activity in adults with scoliosis: what and how much?
What is secondary scoliosis?
Is there a link between scoliosis and flat feet?
How to accept a treatment that “gets in the way”, like back bracing?
Bracing: is it normal to feel scared?
What is scapular winging?
Scoliosis: what positions to sleep in?
Will a scoliosis curve of around 30° get worse or give me back pain during pregnancy?
When scoliosis is just posture?
Up to what age can scoliosis get worse?
Can scoliosis get worse in spite of bracing treatment?

Can adult scoliosis get worse?

13. Https://treatingscoliosis.com/blog/scoliosis-surgery-faqs-get-essential-answers/
What causes scoliosis in children and teens?
How is scoliosis diagnosed and monitored over time?
What are the goals of scoliosis surgery?
What happens during scoliosis fusion surgery?

14. Https://www.globusmedical.com/pediatrics/scoliosis-faq/
What is scoliosis?
What are the different types of pediatric scoliosis?
What causes scoliosis?
Is scoliosis genetic/hereditary?
How common is scoliosis?
What are the symptoms of scoliosis?
How is scoliosis diagnosed?
How is scoliosis treated?

14. https://scoliosis3dc.com/scoliosis-resources/frequently-asked-questions/
What Types of Spinal Curves Do We Treat?
Who is a Candidate for Our Schroth Method Treatment?
Why would a post-surgical patient want Schroth Therapy?
Who is a Candidate for our Scoliosis Bracing?
How Long Does Treatment at Scoliosis 3DC Take?

15. https://scolicare.com/patients-scoliosis-faqs/
How is scoliosis diagnosed?
Which scoliosis do | have?
I have been diagnosed with scoliosis and told to ‘wait and see’. What should | do?
Scoliosis - when to brace scoliosis and where to go?
Who treats scoliosis in adults?
Are scoliosis braces effective?
What is the best treatment for scoliosis?
If I have scoliosis, will my children have it?
Why didn’t we recognise the scoliosis sooner?
Can exercise fix my scoliosis?
How does a program work?
How often should practice occur?

16. https://spineandscoliosis.com/scoliosis-surgery-faq/
How many days will I be in the hospital?
How long will my surgery take?
How soon will I notice improvement?
When can I shower after my surgery?
Will I have to wear a brace after my surgery?
When can I go back to school/work and sport/activities?
How is pain managed after surgery?

How can I best prepare for ASC surgery?
Who should consider Anterior Scoliosis Surgery (ASC)?
How long will I have to wait to get my surgery done?

17. https://www.srs.org/Files/Patient-Brochures/Patient_AIS_Surgery-handbook_FAQ.pdf
What should I bring with me to the hospital?
Do I need to do anything special before my surgery?
Should I take my regular medications before surgery?
What if I am having my period at the time of surgery?
What is done during the surgery?
Who will be in the operating room with me?
Can parents come into the operating room?
Will I wake up during the surgery?
Will I require a blood transfusion as a result of the surgery?
Is it possible to get my own blood back if I need a blood transfusion?
Do I need a plastic surgeon to close my incision?
