# Supplementary material for: An Assessment of GPT-3.5 and GPT-4.0 Responses to Scoliosis FAQs
Source: J Pers Med. 2026 Apr 7;16(4):206. doi: 10.3390/jpm16040206 (PMC13117403; doi:10.3390/jpm16040206)
Supplement: Supplementary file 1 [file jpm-16-00206-s001.zip › Table S2_ChatGPT3.5_Responses.pdf]

<u>Legend</u>

Note that all responses are verbatim with minimal formatting.

Bold text = common responses across six repeats of the questions

Blue-highlighted text = unique responses across six repeats of the question

Yellow-highlighted text = recommends consultation with a healthcare professional

Referral Score: the number of times a referral to a healthcare professional is made, across six times

**GPT-3.5**	**3.5.1-Repeat**	**3.5.2-Repeat**	**3.5.3-Repeat**	**3.5.4-Repeat**	**3.5.5-Repeat**	**RefS.**
Of course, I'd be happy to answer your questions about scoliosis:						
**Q1: What is scoliosis?**<br><br>Scoliosis is a medical condition characterized by an abnormal lateral (sideways) curvature of the spine. **This curvature can be in the shape of an "S" or a "C" an**d may develop in various degrees of severity. **It often occurs during growth spurts in childhood or adolescence but can also result from other factors**, and it can affect individuals of all ages.	Scoliosis is an abnormal sideways curvature of the spine, **often forming an "S" or "C" shape when viewed from the front or back.** It can develop for various reasons, including idiopathic causes with no clear underlying reason, congenital factors, neuromuscular conditions, or degeneration of spinal discs and joints with age. Common symptoms include uneven shoulders, an uneven waistline, and, in severe cases, a noticeable curvature of the spine.	Scoliosis is a medical condition characterized by an **abnormal sideways curvature of the spine, often forming an "S" or "C" shape.** It can result from various factors, including genetic, congenital, neuromuscular, or degenerative causes, and it can affect individuals of all ages. Treatment options vary based on factors like the degree of curvature, age, and underlying cause, ranging from observation and non-surgical methods to surgical intervention.	Scoliosis is an **abnormal sideways curvature of the spine, often forming an "S" or "C" shape** when viewed from the front or back. It **can develop at any age but is most common in adolescents** and can vary in severity. Management depends on factors like age and the degree of curvature and may include observation, bracing, physical therapy, or surgery.	Scoliosis is a medical condition characterized by an **abnormal lateral or rotational curvature** of the spine. **It can occur at any age but is most commonly diagnosed in adolescents during periods of growth**. While mild cases may have no noticeable symptoms, severe scoliosis can lead to physical deformity, back pain, and potential respiratory issues.	Scoliosis is a medical condition characterized by an abnormal sideways curvature of the spine. **It can occur at any age but is often detected during adolescence**. The severity varies, and treatment options range from observation and physical therapy for mild cases to bracing and surgery for more severe cases.	
Median rating = 3.0<br>STD = 0.14						**0.0**
**Q2: How can I prevent scoliosis?**<br><br>Scoliosis is **primarily a structural issue of the spine**, and there **are no known ways to prevent it through lifestyle choices or behaviors.** Some cases of scoliosis have genetic or hereditary components, making prevention impossible. **Regular**	Scoliosis, especially idiopathic scoliosis (the most common type), is generally **not preventable through specific actions or lifestyle changes,** as its exact cause remains unclear. However, maintaining a healthy lifestyle, practicing good posture, and **early**	Scoliosis **cannot be entirely prevented as its causes are often unknown or genetic**. However, **maintaining a healthy lifestyle with regular exercise, proper nutrition, and good posture can support** spine health. **Early detection through**	Scoliosis **cannot be entirely prevented, as its exact cause is often unknown.** However, **maintaining good posture, staying physically active**, and **attending regular check-ups with a healthcare provider can help monitor spine**	Preventing scoliosis is challenging as it is often influenced by genetic and congenital factors. However, **maintaining a healthy lifestyle with regular exercise, good posture habits,** and **early detection through regular check-ups** can help minimize the risk of	Unfortunately, there is **no known way to prevent idiopathic scoliosis, the most common form** that typically develops during adolescence. However, you can support overall spinal health by **maintaining good posture, staying physically active, and**	

GPT-3.5	3.5.1-Repeat	3.5.2-Repeat	3.5.3-Repeat	3.5.4-Repeat	3.5.5-Repeat	RefS.
**medical check-ups and early detection, however, can help manage the condition more effectively if it does develop.**	**detection through regular check-ups can contribute to better spine health and potentially improve the chances of effective management if scoliosis does develop.** Early intervention and appropriate medical care are essential for addressing scoliosis effectively and preventing further deterioration of the spinal curvature.	**regular medical check-ups is crucial for timely intervention and management if scoliosis develop**s.	**development and detect scoliosis early** for timely intervention if needed.	complications associated with scoliosis and ensure timely management if the condition develops.	**performing spine-strengthening exercises. Regular check-ups with a healthcare provider can help detect scoliosis early,** allowing for timely intervention if needed.	
Median rating = 2.0<br>STD = 1.15						**6/6 =1.0**
**Q3: Can scoliotic curves progress?**<br><br>**Yes, scoliotic curves can progress, especially during periods of rapid growth, such as adolescence.** The degree of progression varies from person to person and depends on factors like the type of scoliosis, the age of onset, and treatment interventions. Regular monitoring by a healthcare professional is essential to track curve progression.	**Yes, scoliotic curves can progress, especially during periods of rapid growth, such as adolescence**. The progression of scoliosis varies depending on factors like the underlying cause, age of onset, and severity of the curvature. Early detection and timely intervention are crucial to manage scoliosis effectively and potentially prevent further progression of the spinal curvature.	**Yes, scoliotic curves can progress, especially during periods of rapid growth, such as adolescence**. The progression of scoliosis depends on factors like age, the degree of curvature, and the underlying cause. Monitoring by a healthcare professional is essential to assess and manage potential progression effectively.	**Yes, scoliotic curves can progress, especially during periods of growth and development, like adolescence.** The extent of progression depends on factors such as age, the degree of curvature, and the underlying cause of scoliosis. Regular monitoring and early intervention are crucial in managing scoliosis effectively and preventing further progression.	**Yes, scoliotic curves can progress, particularly during periods of growth and development, such as adolescence.** The rate and extent of progression can vary, making regular monitoring and early intervention crucial to prevent scoliosis from worsening.	**Yes, scoliotic curves can progress, especially during periods of growth, such as adolescence.** The progression of scoliosis depends on factors like the individual's age, the initial degree of curvature, and the potential for further growth. Monitoring and intervention are essential to manage and potentially slow down the progression of scoliosis.	
Median rating = 2.0<br>STD = 1.07						**6/6 =1.0**
**Q4: Do I need scoliosis surgery?**<br><br>Whether or not you need scoliosis surgery depends on **several factors, including the severity of your spinal curvature, your age, and**	Whether you need scoliosis surgery depends on **factors like the severity and progression of your condition, your age, and the presence of symptoms or complications.** Surgery is	Whether you need scoliosis surgery depends on **factors like the degree of curvature, your age, the underlying cause of scoliosis, and how the condition affects your daily life.** Surgery is	The need for scoliosis surgery is determined on an individual basis and depends on **factors like the severity of the spinal curvature, the presence of symptoms, and the age of the individual.**	The need for scoliosis surgery depends on **factors such as the severity of the spinal curvature, the progression of the condition, the age of the individual, and the**	Whether you need scoliosis surgery depends on **factors like the severity of the spinal curvature, the potential for progression, and the impact on your health and quality of life.** It is a	

GPT-3.5	3.5.1-Repeat	3.5.2-Repeat	3.5.3-Repeat	3.5.4-Repeat	3.5.5-Repeat	RefS.
**whether non-surgical interventions have been effective in managing the condition**. Surgical intervention is usually considered when the curvature becomes severe (**typically over 40-50 degrees) and is accompanied by other factors like pain, breathing difficulties, or functional limitations.**	typically considered when other treatments have not effectively controlled the progression of scoliosis and the curvature has become severe or is causing significant health issues. The decision to undergo surgery is made in consultation with a healthcare provider who evaluates your specific case and discusses the potential benefits and risks associated with surgery.	typically considered for severe curvature or when non-surgical treatments are ineffective or if scoliosis causes significant pain, disability, or complications. The decision should be made in consultation with a healthcare specialist who can provide personalized recommendations based on your specific circumstances.	Surgery is typically considered when non-surgical treatments have not been effective, and the curvature is severe or significantly impacts the person's quality of life. A healthcare provider or orthopedic specialist can assess your specific case and recommend surgery if it is deemed necessary.	**underlying cause**. Surgery is typically considered when non-surgical treatments are ineffective, and the benefits of surgery outweigh the risks and potential complications. The decision should be made in consultation with a healthcare professional, such as an orthopedic specialist, who can assess the individual's specific case and provide personalized guidance.	decision that should be made in consultation with a healthcare provider or orthopedic specialist who can assess your specific case and provide recommendations based on your individual circumstances. Conservative treatments and regular monitoring are often considered before surgery is recommended for severe or progressing cases.	
Median rating = 3.0<br>STD = 1.07						**5/6 =0.83**
**Q5: How can I avoid scoliosis surgery?**<br><br>**Avoiding scoliosis surgery may be possible through non-surgical interventions if the curvature is detected early and is not too severe.** These interventions may include bracing, physical therapy, and exercises to help stabilize or reduce the curve. Regular follow-up with a spine specialist is essential to monitor progress and adjust the treatment plan as needed.	To potentially avoid scoliosis surgery, **early detection and monitoring are crucial, especially during growth periods. Conservative treatments like bracing or physical therapy may be recommended** to manage and stabilize the spine. Maintaining a healthy lifestyle and engaging in open communication with healthcare providers are essential to consider all available treatment options and potentially delay or avoid surgery.	To potentially avoid scoliosis surgery, **focus on early detection through regular check-ups and timely intervention**. Promote spine health with a healthy lifestyle, including exercise, proper nutrition, and good posture habits. Compliance with **non-surgical treatments and physical therapy can help manage scoliosis effectively**, and early monitoring is key if the condition develops.	Avoiding scoliosis surgery involves measures such as **early detection through regular check-ups, exploring non-surgical treatments like physical therapy and bracing,** maintaining a healthy lifestyle, monitoring curvature progression, and managing pain or discomfort through conservative methods. The decision to undergo surgery is highly individualized and is typically made when non-surgical treatments are ineffective, the curvature is severe, or there is a significant impact on the individual's quality of life. Consulting with healthcare providers and orthopedic specialists is crucial to determine the	To avoid scoliosis surgery, **early detection and proactive management** are key. Regular check-ups with a healthcare provider, adherence to **non-surgical treatments like bracing and physical therapy when necessary, and maintaining a healthy lifestyle** can help prevent scoliosis from progressing to the point where surgery is needed. Seeking expert medical advice and a second opinion if recommended surgery is important for making informed decisions about treatment options.	To potentially avoid scoliosis surgery, **early detection through regular check-ups is crucial**, especially during growth periods in children and adolescents. If scoliosis is detected, following recommended **conservative treatments, maintaining a healthy lifestyle, and seeking regular monitoring by healthcare providers can help manage the condition effectively and potentially prevent the need for surgery in some cases**. Consulting with healthcare professionals for personalized guidance and adhering to treatment plans are essential steps in avoiding surgery when possible.	

GPT-3.5	3.5.1-Repeat	3.5.2-Repeat	3.5.3-Repeat	3.5.4-Repeat	3.5.5-Repeat	RefS.
			most suitable treatment plan.			
Median rating = 2.0<br>STD = 1.15						**5/6 =0.83**
**Q6: What sports can I do with scoliosis?**<br><br>Many individuals with scoliosis can participate in sports and physical activities**. The choice of sports will depend on the severity of the scoliosis, the level of pain or discomfort, and the advice of your healthcare provider**. Low-impact sports like swimming and yoga are often recommended, but it's essential to consult with a healthcare professional for personalized guidance.	Individuals with scoliosis can often participate in a wide range of sports and physical activities, but it depends on factors like the severity of the condition and individual preferences. Low-impact activities like swimming, yoga, and walking are generally well-tolerated, while high-impact or contact sports should be approached with caution. Consulting with healthcare providers and specialists can help determine which sports and activities are safe and suitable for your specific case.	Individuals with scoliosis can engage in a wide range of sports and physical activities, depending on the severity of their condition and individual comfort. Options include swimming, yoga, walking, cycling, golf, and team sports. Consultation with a healthcare provider or physical therapist can provide guidance on suitable activities and any necessary modifications.	Individuals with scoliosis can generally participate in a wide range of sports and physical activities, but the choice should consider factors like the severity and flexibility of the spinal curvature, symptoms, and healthcare provider recommendations. Low-impact activities like swimming, walking, and yoga are often well-suited, and some higher-impact sports may still be possible with caution and supervision. Consulting with a healthcare provider or orthopedic specialist is crucial to determine which sports and activities are most suitable for your specific condition and health status.	Individuals with scoliosis can often participate in a variety of sports and physical activities, including swimming, yoga, walking, cycling, golf, and more. The choice of sport should consider the severity of the condition and individual comfort levels, with a focus on maintaining spinal health and avoiding activities that may exacerbate pain or discomfort. It's essential to consult with a healthcare provider for personalized guidance on suitable sports and exercises based on the specific case of scoliosis.	Individuals with scoliosis can often participate in a variety of sports and physical activities, but the choice of activities should consider factors like the severity of the curvature and individual comfort. Low-impact activities like swimming, yoga, walking, and cycling are generally safe and beneficial. Consult with a healthcare provider or orthopedic specialist for personalized recommendations based on your specific case and limitations.	
Median rating = 3.0<br>STD = 1.12						**6/6 =1.0**
**Q7: What causes scoliosis?**<br><br>The exact causes of scoliosis can vary. **Some cases are idiopathic, meaning the cause is unknown.** Other potential causes include **genetics, neuromuscular conditions, congenital spine abnormalities, or traumatic injuries.** Understanding the cause of scoliosis in a specific individual often requires a thorough	Scoliosis can have various causes and is categorized into different types based on its underlying origin. **Idiopathic scoliosis, the most common type, often develops without a known cause,** while **congenital scoliosis** results from developmental abnormalities in the vertebrae.	Scoliosis can have various causes, including **idiopathic (unknown), congenital (present at birth), neuromuscular conditions** (such as cerebral palsy or muscular dystrophy), **degenerative changes in the spine, trauma, infections, tumors, and connective tissue disorders. Idiopathic scoliosis is the**	Scoliosis can have various causes depending on its type**. Idiopathic scoliosis, the most common form,** often develops without a known cause, though **genetics** may play a role. Other types include **congenital scoliosis (present at birth due to abnormal spinal development), neuromuscular scoliosis**	The exact cause of scoliosis is often unknown and is referred to as **idiopathic scoliosis,** which is the most common type, particularly among adolescents. While genetics likely play a role in its development, the precise cause remains unclear. **Other forms of scoliosis have known causes, including**	Scoliosis can have various causes, with the most common being **idiopathic scoliosis,** which often develops during adolescence without any identifiable cause. **Congenital scoliosis results from abnormal** vertebral development at birth, while **neuromuscular scoliosis is associated** with	

GPT-3.5	3.5.1-Repeat	3.5.2-Repeat	3.5.3-Repeat	3.5.4-Repeat	3.5.5-Repeat	RefS.
evaluation by a healthcare provider.	**Neuromuscular scoliosis** is associated with neuromuscular conditions, degenerative scoliosis is related to aging and degeneration of spinal structures, and **syndromic scoliosis** is linked to certain genetic syndromes. **Secondary scoliosis can result from factors like trauma, infections, or tumors affecting the spine.**	**most common type,** often occurring during adolescence with no known cause, but it may have a genetic component. The specific cause of scoliosis varies among individuals.	**(associated with neuromuscular conditions), and degenerative scoliosis** (related to age and spinal degeneration).	**congenital scoliosis (present at birth), neuromuscular scoliosis (linked to neuromuscular conditions), and degenerative scoliosis** (associated with age-related changes in the spine).	conditions like cerebral palsy or muscular dystrophy. Other, less common causes include spinal **tumors or injuries**. Many cases of scoliosis remain idiopathic, meaning the exact cause is unknown.	
Median rating = 2.0<br>STD = 1.07						**1/6 =0.17**
**Q8: What are common symptoms of scoliosis?**<br><br>Common symptoms of scoliosis may include an **uneven shoulder or hip height, an asymmetrical appearance of the back when bending forward, and in some cases, back pain or discomfort**. However, scoliosis is not always accompanied by noticeable symptoms, especially in mild cases.	Common symptoms of scoliosis include uneven shoulders, an **uneven waistline, and, in severe cases, a noticeable sideways curvature of the spine. Back pain or discomfort may also occur, especially when the curvature is significant or leads to muscle strain.** It's important to note that scoliosis can develop without noticeable symptoms, particularly in its early stages.	Common symptoms of scoliosis include **uneven shoulder and hip heights, an uneven waistline, a visible "S" or "C" curve in the spine when viewed from the back, a rib hump when bending forward, back pain, and, in severe cases, breathing difficulties.** However, many individuals with scoliosis may not experience noticeable symptoms, especially in the early stages. Early detection through medical evaluation is crucial for diagnosis and management.	Common symptoms of scoliosis may include **uneven shoulders or waist, an asymmetrical ribcage, an uneven appearance when bending forward, back pain, and in severe cases, breathing difficulties due to spinal curvature affecting lung function.** However, not everyone with scoliosis experiences symptoms, and they become more noticeable as the curvature progresses. Seeking evaluation by a healthcare provider or orthopedic specialist is advisable if these signs or discomfort occur.	Common symptoms of scoliosis can include **uneven shoulders, hips, or waist, an asymmetrical ribcage, visible back curvature when bending forward, muscle imbalances, back pain (especially in more severe cases), and, in severe cases, respiratory issues due to changes in the chest cavity**. However, not everyone with scoliosis experiences noticeable symptoms, and the severity of symptoms can vary widely among individuals. Regular check-ups with a healthcare provider, particularly an orthopedic specialist, are important for early detection and monitoring.	Common symptoms of scoliosis include an uneven **appearance of the shoulders, waist, and hips, as well as a visible sideways curvature of the spine when viewed from the front or back. Some individuals may experience back pain, muscle imbalances, reduced flexibility, and, in severe cases, breathing difficulties due to chest compression.** However, it's important to note that not everyone with scoliosis experiences noticeable symptoms, especially in mild cases. Regular check-ups with a healthcare provider can help with early detection and monitoring.	
Median rating = 2.0<br>STD = 0.9						**4/6 =0.67**

GPT-3.5	3.5.1-Repeat	3.5.2-Repeat	3.5.3-Repeat	3.5.4-Repeat	3.5.5-Repeat	RefS.
**Q9: How dangerous is scoliosis surgery?**<br><br>Scoliosis surgery is generally considered **safe when performed by experienced surgeons in appropriate candidates**. Like any surgical procedure, there are risks, including **infection, bleeding, complications with anesthesia, and, in rare cases, neurological or vascular complications**. The decision to undergo surgery should be made after a thorough evaluation and discussion of potential risks and benefits with a healthcare provider.	Scoliosis surgery is generally **considered safe when performed by experienced surgeons, but like any surgical** procedure, it carries risks and potential complications. These can include **infection, blood loss, neurological complications, hardware-related issues, fusion problems, and** anesthesia-related complications. The decision to undergo surgery is made after a thorough evaluation of the individual's condition and a consideration of potential benefits versus risks, **with an emphasis on selecting a skilled surgical team to minimize risks.**	Scoliosis surgery, like any major surgical procedure, carries risks and **potential complications, including infection, bleeding, nerve damage, and hardware-related issues**. However, the level of risk varies depending on individual factors and the **surgeon's experience.** The decision to undergo scoliosis surgery should be made after thorough consultation with healthcare professionals, taking into account the potential risks and **benefits in the context of the individual's unique condition.**	Scoliosis surgery, like any surgical procedure, carries certain risks and potential complications, including **infection, bleeding, nerve or spinal cord injury (though rare), hardware-related issues, postoperative pain, reduced flexibility, and non-union in the fused vertebra**e. However, the decision to undergo surgery is typically made when the benefits outweigh the risks, especially in cases of severe scoliosis where non-surgical treatments have been ineffective or when the condition significantly impacts a person's quality of life. Surgeons work to minimize risks, and rehabilitation plays a crucial role in the recovery process to regain function and mobility.	Scoliosis surgery carries inherent risks and potential complications, but its safety and effectiveness depend on factors such as the individual's overall health, the type of surgery, and the degree of spinal correction needed. Some potential risks include **infection, bleeding, nerve damage, hardware complications, and pain.** However, surgery is generally **considered safe and effective when performed by experienced surgeons** in specialized centers, and the decision to undergo surgery is typically made when the benefits outweigh the risks, especially in cases of severe scoliosis or when non-surgical treatments have not been effective.	Scoliosis surgery can be effective in treating the condition, but it carries some potential risks and complications, **including infection, blood loss, nerve damage, hardware-related issues, post-operative pain, and complications related to anesthesia.** These risks are typically managed by experienced surgeons and medical teams, and the decision to undergo surgery should be made in consultation with a qualified medical professional, taking into account the individual's specific circumstances and the potential benefits of the surgery. While there are risks involved, scoliosis surgery has helped many individuals achieve improved spinal alignment and quality of life.	
Median rating = 2.0<br>STD = 0.58						**3/6 =0.5**
**Q10: What movements will I be able to do after scoliosis surgery?**<br><br>The ability to move after **scoliosis surgery depends on the surgical technique used, the location and extent of the spinal fusion, and individual**.	The extent of movements after scoliosis surgery depends on factors like the **surgical approach, level of spinal fusion, and individual healing**. While spinal fusion can limit the flexibility of the fused portion of the spine, individuals typically maintain the ability to perform everyday	After scoliosis surgery, the ability to perform movements will depend on factors such as the **surgical approach, the extent of spinal correction, and individual recovery.** While there may be some restrictions on spinal mobility in the areas that were fused, **physical**	The extent of movements you can perform after **scoliosis surgery depends on factors such as the type of surgery and the number of vertebrae fused.** While the fused portion of the spine may have reduced flexibility, you **should still be able to perform most activities of daily living, and**	After scoliosis surgery, your **ability to move and perform various activities will depend on factors such as the type of surgery, the degree of spinal correction achieved, and your overall health.** While there may be some limitations in spinal mobility, many individuals	The movements you can **perform after scoliosis surgery depend on factors like the surgical technique used and the extent of spinal correction**. While there may be some limitations in spinal range of motion, most individuals can **still perform activities of daily living such as**	

GPT-3.5	3.5.1-Repeat	3.5.2-Repeat	3.5.3-Repeat	3.5.4-Repeat	3.5.5-Repeat	RefS.
	activities such as walking, sitting, and standing. Participation in sports and physical activities may be possible, depending on the level of fusion and individual circumstances, with guidance from your surgeon and appropriate rehabilitation.	**therapy and rehabilitation can help regain flexibility, range of motion, and strength.** The goal is to improve spinal alignment and stability while preserving as much mobility as possible in adjacent spinal segments, with open communication with healthcare providers guiding your postoperative movements and activities.	**physical therapy can help you regain strength and mobility.** The range of motion and flexibility in the upper and lower spine, away from the fused area, should generally remain relatively unaffected.	experience improved posture, reduced pain, and better overall function. The specific movements and activities you can engage in after surgery will be discussed with your healthcare team, and a tailored rehabilitation program will help you regain strength and flexibility while minimizing the risk of complications.	**walking, sitting, and standing. Physical therapy and rehabilitation are crucial for gradually regaining mobility and strength, and many individuals can eventually return to physical activities and exercise routines**, with some caution and guidance, as part of their recovery process.	
Median rating = 3.0<br>STD = 0.69						**3/6 =0.5**
Remember that these answers are general in nature and it's important to consult with a qualified medical professional for personalized advice and recommendations based on your specific situation.						
