# Supplementary material for: An Assessment of GPT-3.5 and GPT-4.0 Responses to Scoliosis FAQs
Source: J Pers Med. 2026 Apr 7;16(4):206. doi: 10.3390/jpm16040206 (PMC13117403; doi:10.3390/jpm16040206)
Supplement: Supplementary file 1 [file jpm-16-00206-s001.zip › Table S3_ChatGPT4.0_Responses.pdf]

Legend

Note that all responses are verbatim with minimal formatting.

Bold text = common responses across six repeats of the questions

Blue-highlighted text = unique response across six repeats of the question

Yellow-highlighted text = recommends consultation with a healthcare professional

Referral Score (RefS.): number of times a referral to a healthcare professional is made across six times

**GPT-4.0**	**4.01-Repeat**	**4.02-Repeat**	**4.03-Repeat**	**4.04-Repeat**	**4.05-Repeat**	**RefS.**
I'm not a healthcare professional, but I can provide you with some general information on scoliosis. If you're concerned about this condition, it's essential to consult with a medical doctor for accurate diagnosis and appropriate treatment options tailored to your needs.						
**Q1: What is scoliosis?**<br><br>**Scoliosis is a medical condition where there is an abnormal lateral (sideways) curvature of the spine**. When viewed from the back, a typical spine is straight. In a person with scoliosis, the **spine can curve to the left, right, or both, often resembling an "S" or "C"** shape.	**Scoliosis is a condition where the spine curves sideways in an "S" or "C" shape**, most commonly diagnosed in children and adolescents. The cause is often unknown, but it can be associated with genetic, neuromuscular, or congenital factors. Treatment varies from monitoring to bracing or surgery, depending on the curve's severity and progression.	**Scoliosis is a medical condition characterized by an abnormal sideways curvature of the spine**, often manifesting **in "S" or "C" shapes.** While its exact cause is frequently unknown, it can be linked to genetic, neuromuscular, or congenital factors. Treatment options range from observation and bracing to surgery, depending on the severity and progression of the curvature.	Scoliosis is a **condition where the spine curves sideways in an "S" or "C" shape, deviating from a normal straight line**. It most commonly occurs during the growth spurt before puberty. The severity and treatment vary, ranging from monitoring to bracing or, in severe cases, surgery.	Scoliosis is a **condition where the spine curves sideways in an "S" or "C" shape, differing from the normal vertical alignment**. It most commonly arises during childhood or adolescence, and its severity can range from mild to severe. The cause of most cases is unknown, though it can also stem from congenital, neuromuscular, or degenerative issues.	Scoliosis is a **condition where the spine curves sideways, often forming an "S" or "C" shape, rather than a straight line**. This condition most commonly develops during childhood or adolescence, with the exact cause often unknown. Symptoms can include uneven shoulders or hips, and treatment varies from observation to bracing or surgery, depending on the severity.	
Median rating = 3.0<br>STD = 0.31						**0.0**
**Q2: How can I prevent scoliosis?**<br><br>**Many cases of scoliosis are idiopathic, meaning the cause is unknown**, so	Scoliosis, particularly its most common form, adolescent idiopathic scoliosis, cannot be **prevented as its exact causes are largely**	Preventing scoliosis is challenging since most cases are **idiopathic with unknown causes, and not directly linked to preventable factors.**	Preventing scoliosis, particularly its most common form, **idiopathic scoliosis, is challenging as the exact causes are often unknown.**	Preventing scoliosis is challenging as **most cases are idiopathic, meaning their cause is unknown and not linked to specific preventable factors**.	Preventing scoliosis is **challenging, as many cases, particularly idiopathic scoliosis, have unknown causes and may involve genetic**	

GPT-4.0	4.01-Repeat	4.02-Repeat	4.03-Repeat	4.04-Repeat	4.05-Repeat	RefS.
prevention is not always possible. **However, early detection and appropriate management can prevent progression in some cases. Regular check-ups during growth spurts in children can be helpful.**	**unknown** and believed to be a mix of genetic and environmental factors. The best approach is **early detection and management, which includes regular health screenings during childhood and adolescence.** Maintaining good overall health, including bone health and muscular strength, may also be beneficial.	However, maintaining overall back health with good posture, a balanced diet, and regular exercise can be beneficial**. It's also important to have regular check-ups for early detection, especially if there's a family history of scoliosis.**	However, maintaining good overall back health through regular exercise, a balanced diet rich in calcium and vitamin D, and monitoring children's spinal development during growth spurts can be helpful. **Early detection and management are key to addressing scoliosis effectively if it develops.**	However, 0 overall spine health through regular exercise, good posture, and a balanced diet can be beneficial. **Early detection through regular health check-ups, especially during childhood and adolescence, is key for timely intervention and management**.	**factors.** Maintaining good overall back health through regular exercise, proper posture, and ergonomic practices can contribute to spinal health, but these measures do not guarantee prevention of scoliosis. **Early detection and management are key, especially during childhood growth spurts, to monitor and address any spinal curvature that may develop**.	
Median rating = 2.0<br>STD = 0.90						**4/6 =0.7**
**Q3: Can scoliotic curves progress?**<br><br>**Yes, scoliotic curves can progress**, especially during **rapid growth periods in adolescence. Regular monitoring** by a healthcare professional is important to determine the **progression and decide on appropriate treatment**.	**Yes, scoliotic curves can progress**, particularly during periods **of rapid growth like adolescence**. The likelihood of progression depends on factors such as the severity of the curve, the age at diagnosis, and the individual's growth potential. **Regular monitoring and treatment, when necessary, are key to managing this progression.**	**Yes, scoliotic curves can progress, particularly during periods of rapid growth like adolescence**. The likelihood of progression depends on factors such **as the size of the curve at diagnosis and the patient's age.** Regular **monitoring and early intervention are key in managing scoliosis and preventing further curvature.**	**Yes, scoliotic curves can progress, especially during periods of rapid growth such as adolescence**. The risk of progression is influenced by factors **like the severity of the curve at initial diagnosis, age, and the specific type of scoliosis**. **Regular monitoring and appropriate treatment are essential to manage and potentially mitigate progression.**	**Scoliotic curves can progress, particularly during periods of rapid growth such as adolescence**. The likelihood of progression depends on factors like the size of the curve and the age at onset. **Regular monitoring and appropriate treatment, such as bracing, are crucial to manage and potentially prevent further progression.**	**Scoliotic curves can indeed progress, particularly during periods of rapid growth such as adolescence**. The likelihood of progression depends on factors like the severity of the curve and the patient's age. **Regular monitoring and appropriate interventions, like bracing or surgery, are crucial in managing and potentially mitigating this progression**.	
Median rating = 2.8<br>STD = 0.90						**6/6 =1**
**Q4: Do I need scoliosis surgery?**<br><br>The decision for scoliosis surgery is based on several factors, including the **severity and location of the curve, the patient's**	**Determining the need for scoliosis surgery depends on individual factors like the severity of the spinal curve, its rate of progression, and the presence of symptoms such as pain or**	**Determining the need for scoliosis surgery depends on various factors, including the severity and progression of the spinal curve, the presence of symptoms, and the response to non-**	**Whether you need scoliosis surgery depends on several factors, including the severity and progression of the spinal curvature, the presence of symptoms like pain or breathing**	**Whether you need scoliosis surgery depends on several factors, including the severity and progression of the curve, associated symptoms, and overall impact on your quality**	**Whether you need scoliosis surgery depends on various factors, including the severity and progression of your spinal curvature, your age, and any associated symptoms like pain or**	

GPT-4.0	4.01-Repeat	4.02-Repeat	4.03-Repeat	4.04-Repeat	4.05-Repeat	RefS.
**age, and the risk of progression**. **Generally, surgery is considered for curves that are greater than 40-50 degrees** or if the curve is progressing despite other treatments. An orthopedic surgeon specializing in spine disorders can provide guidance.	**functional impairment.** Consultation with a healthcare professional, typically an orthopedic surgeon, is essential to evaluate these factors and decide if surgery is the appropriate course of action.	**surgical treatments**. A thorough evaluation by a healthcare professional, usually a spine specialist, is essential to assess whether surgery is the appropriate course of action for your specific case.	**difficulties, and your overall health.** This decision should be made in consultation with healthcare professionals, such as an orthopedic surgeon or spine specialist, who can evaluate your specific case and recommend the best treatment plan.	**of life. Surgery is typically considered for more severe cases, often when curves exceed 45 to 50 degrees or when non-surgical treatments like bracing are ineffective**. It's a decision made in consultation with healthcare professionals specializing in spinal disorders.	**functional impairment**. Surgery is typically considered for more severe cases (curves greater than 45-50 degrees) or when non-surgical treatments have not effectively managed the condition. A thorough evaluation by a healthcare professional is essential to determine the best course of action for your specific situation.	
Median rating = 2.5<br>STD = 1.26						**6/6 =1.0**
**Q5: How can I avoid scoliosis surgery?**<br><br>**Non-surgical treatments, like bracing or physical therapy, can sometimes help manage scoliosis and prevent its progression, particularly in mild to moderate** cases. Adherence to prescribed treatments and regular follow-ups are crucial.	To avoid scoliosis surgery, focus on early detection and consistent management of the condition, including using **a brace if prescribed, especially during growth periods. Engaging in physical therapy and exercises to strengthen back muscles, maintaining a healthy lifestyle**, and regular monitoring of the spine's curvature with a healthcare professional are also key strategies.	To potentially avoid **scoliosis surgery, it's important to manage the condition with non-surgical treatments like bracing, physical therapy, and regular exercise, especially if the spine is still growing**. Close monitoring of the curvature, maintaining a healthy lifestyle, and regular check-ups with a **healthcare provider** can also help in managing the condition effectively and possibly prevent the need for surgery.	To potentially avoid **scoliosis surgery, focus on early detection, regular monitoring, and non-surgical management strategies like physical therapy, bracing (particularly in children and adolescents), and maintaining a healthy lifestyle**. These approaches aim to control the progression of the curvature and manage symptoms, thereby reducing the likelihood of needing surgical intervention. However, the effectiveness of these strategies can vary depending on the individual case and severity of the scoliosis.	To avoid scoliosis **surgery, early detection and proactive management of the condition are crucial. This often involves using a back brace to prevent further curvature progression, especially in growing children and adolescents, and engaging in physical therapy or** specific exercises to strengthen supporting muscles. Regular monitoring by healthcare professionals is essential to adjust treatment plans and assess the curve's progression.	To avoid scoliosis **surgery, early detection and management are key, with non-surgical treatments like bracing often used to prevent further curvature progression in growing individuals**. Regular exercise, maintaining a healthy weight, and monitoring the condition with a healthcare provider can also be beneficial. However, the effectiveness of these methods varies, and surgery may still be necessary in more severe cases.	
Median rating = 2.0<br>STD = 1.15						**5/6 =0.83**

GPT-4.0	4.01-Repeat	4.02-Repeat	4.03-Repeat	4.04-Repeat	4.05-Repeat	RefS.
**Q6: What sports can I do with scoliosis?**<br><br>Many people with scoliosis can participate in a wide range of sports. The type and severity of scoliosis, as well as the specific sport in question, will influence this. It's essential to consult with a healthcare provider to determine which activities are safe and beneficial.	Individuals with scoliosis **can participate in many sports, especially low-impact and non-contact activities such as swimming, cycling, and walking, which are generally beneficial for spinal health**. The choice of sport should be based on personal comfort and the avoidance of activities that exacerbate symptoms or the curvature. Consulting with a healthcare provider for personalized advice and any necessary modifications is recommended.	Individuals with scoliosis **can participate in most sports, focusing on low-impact activities that promote flexibility and core strength without excessive strain on the spine. Swimming, cycling, yoga, and Pilates are often recommended due to their supportive nature for back health**. It's important, however, to consult with a healthcare provider before starting any new sport to ensure it aligns with your specific condition.	Individuals with scoliosis can engage in **most sports, particularly those that are low-impact and symmetrical, such as swimming, cycling, and running. Sports that emphasize core strength and flexibility, like yoga and Pilates,** are also beneficial. It's important to consult with a healthcare provider for personalized advice, especially for high-impact or contact sports.	Individuals with scoliosis can engage in a **variety of sports, particularly those that are low-impact and do not strain the spine, such as swimming, cycling, and running.** It's important to consult healthcare providers to choose activities that are safe and beneficial for your specific condition, and modifications may be needed for certain sports to avoid exacerbating the curvature.	Individuals with **scoliosis can usually participate in a variety of sports, especially those that do not put excessive strain on the spine, like swimming, cycling, and running.** It's important to consult with a healthcare provider before engaging in any sports, as the type and severity of scoliosis might necessitate certain modifications or restrictions. Regular physical activity, in fact, can be beneficial for maintaining flexibility and strengthening the muscles supporting the spine.	
Median rating = 3.0<br>STD = 0.94						**6/6<br>=1.0**
**Q7: What causes scoliosis?**<br><br>The exact cause of **scoliosis is unknown in most cases and is termed "idiopathic."** However, scoliosis can be associated with conditions like cerebral palsy, muscular dystrophy, genetic conditions, or birth defects. Some cases may also result from injuries, infections, or tumors.	Scoliosis is often idiopathic, meaning its exact cause is unknown, though it's believed to involve genetic and environmental factors. Other types, less common, can be due to congenital spinal malformations, neuromuscular conditions, degenerative changes in the spine, or as part of a syndrome like Marfan or Ehlers-Danlos.	The exact cause of most **cases of scoliosis, known as idiopathic scoliosis, remains unknown but is believed to involve genetic factors**. Other types can be caused by neuromuscular conditions, congenital spinal deformities, or degenerative changes in the spine. Environmental factors are generally not considered direct causes of scoliosis.	Scoliosis can be caused by factors **such as genetic predisposition (in idiopathic scoliosis, the most common type), congenital spine anomalies, neuromuscular conditions, and degenerative spinal changes.** However, the exact cause of most scoliosis cases remains unknown.	The exact cause of most **scoliosis, known as idiopathic scoliosis, is unknown, although it often appears during the growth spurt before puberty and may have a genetic component**. Other forms can result from congenital spinal malformations, neuromuscular conditions, or degenerative spinal changes, particularly in adults. Environmental factors have not been conclusively linked to its development.	Scoliosis can be **caused by a variety of factors, with the most common form, idiopathic scoliosis, having an unknown cause, potentially involving genetic factors.** Other types include congenital scoliosis, resulting from spinal malformations at birth, and neuromuscular scoliosis, linked to conditions like cerebral palsy. Degenerative scoliosis occurs in adults due to the wear and tear of spinal structures.	
Median rating = 2.2<br>STD = 1.21						**0.0**

GPT-4.0	4.01-Repeat	4.02-Repeat	4.03-Repeat	4.04-Repeat	4.05-Repeat	RefS.
**Q8: What are common symptoms of scoliosis?**<br><br>Symptoms can vary based on **the severity, but common signs include an uneven waist, leaning to one side, asymmetry in shoulder or hip height, and uneven shoulder blades**. Some people may experience back **pain or respiratory issues** in severe cases.	Common symptoms of scoliosis include **a visible curve in the spine, uneven shoulders or waist, one hip higher than the other, and possibly a protruding rib cage or shoulder blade**. In more severe cases, individuals may experience back **pain, reduced flexibility, and in extreme cases, breathing difficulties.**	Common symptoms of **scoliosis include a visible curve in the spine, uneven shoulders or hips, and one shoulder blade that may appear more prominent. In some cases**, individuals might experience **back pain or notice clothes fitting unevenly due to the curvature**. The severity of symptoms can vary based on the degree of the spinal curve.	Common symptoms of scoliosis include a visible curve in the **spine, uneven shoulders, an asymmetrical waist, one hip appearing higher than the other, and in** some cases, **back pain or discomfort. In** severe cases, it can also lead to breathing difficulties due to the altered shape of the rib cage.	Common symptoms **of scoliosis include a noticeable curve in the spine, uneven shoulders or waist, one shoulder blade** protruding more than the other, and in some cases, **back pain or discomfort. In severe cases, it can lead to reduced range of motion and difficulties with breathing due to altered chest cavity structure.**	Common symptoms **of scoliosis include a visible curve in the spine, uneven shoulders or hips, and one shoulder blade that may appear more prominent. In some cases**, individuals may experience back **pain or notice a change in their posture, such as leaning to one side.** The severity of symptoms varies, often depending on the degree of the spinal curvature.	
Median rating = 2.0<br>STD = 1.00						**0.0**
**Q9: How dangerous is scoliosis surgery?**<br><br>All surgeries carry risks. Common risks with scoliosis surgery include **bleeding, infection, nerve damage, and problems with the hardware used to correct the spine**. Modern surgical techniques have made the **procedure safer, but the outcome can vary based on the individual's health, the severity of the scoliosis, and the surgeon's experience**.	Scoliosis surgery, typically spinal fusion, is a major procedure with risks like any significant surgery, **including complications from anesthesia, infection, blood loss, and potential nerve damage**. However, advances in surgical **techniques and postoperative care have significantly improved its safety and efficacy**. The decision to undergo surgery should be made after thorough consultation with healthcare professionals, considering individual health factors and the severity of the condition.	Scoliosis surgery, like any major surgical procedure, carries risks **including infection, blood loss, and potential nerve damage, but advancements in surgical techniqu**es have significantly **improved safety and outcomes**. The decision to undergo surgery depends on individual factors such as the **severity and progression of the curve, and it's typically considered when non-surgical treatments are ineffectiv**e. Regular follow-up and adherence to rehabilitation post-surgery are crucial for recovery.	Scoliosis surgery, like any major surgery, **carries risks including infection, bleeding, and potential nerve damage, but advances in surgical techniques have significantly improved its safety.** The decision to undergo surgery involves weighing these risks against the **potential benefits of pain relief and improved spinal alignment, and it should be made in consultation with a healthcare** professional.	Scoliosis surgery, typically involving spinal fusion, is a **major procedure with risks like infection, nerve damage, and complications from anesthesia, but it is generally considered safe with modern surgical techniques**. The level of risk varies based on the **individual's health and the complexity of the surgery, and long-term outcomes are usually positive, significantly improving** quality of life for those with severe scoliosis.	Scoliosis surgery, such as spinal fusion, carries risks like any **major surgery, including infection, bleeding, and potential nerve damage.** However, with advancements in surgical techniques, the safety and outcomes have significantly improved. The decision to undergo surgery should involve a thorough discussion of these risks with a healthcare provider, balanced against the potential benefits.	

GPT-4.0	4.01-Repeat	4.02-Repeat	4.03-Repeat	4.04-Repeat	4.05-Repeat	RefS.
Median rating = 3.0<br>STD = 0.90						**3/6 = 0.5**
**Q10: What movements will I be able to do after scoliosis surgery?**<br><br>Post-surgical mobility varies depending on the type of surgery and the individual's recovery. **Initially, movements might be limited to ensure proper healing. Over time, and with physical therapy, many people regain significant mobility and can engage in everyday activities.** Some high-impact activities or extreme spinal motions **might be restricted permanently or for an extended period after surgery.**	After scoliosis surgery, particularly spinal fusion, your range of motion may be limited, **especially in the area of the spine that was operated on**. The extent of this limitation depends on factors like the **location of the fusion and the number of vertebrae fused.** Over time, with physical therapy and healing, **you'll likely regain strength and some degree of flexibility, adapting to the new range of motion**.	After scoliosis surgery, particularly spinal fusion, **your range of motion may be limited in the area where the vertebrae are fused.** This limitation varies depending on the location and extent of the fusion, **with potential restrictions in bending, twisting, or turning.** Physical therapy post-surgery plays a critical role in helping regain strength and learn how to adapt to these new movement patterns.	After scoliosis surgery, your range of **motion may be somewhat limited, particularly in the area of the spine that was fused.** Initially, movements like bending, twisting, or lifting heavy objects will be restricted to allow healing. **Over time, with physical therapy, you'll likely regain a significant degree of mobility, although some** permanent limitations may remain depending on the extent of the fusion.	After scoliosis **surgery, your range of spinal movement will likely be reduced, particularly in areas where the spine has been fused.** The extent of this limitation depends on the specific regions of the spine involved in the surgery. **Postoperative physical therapy is crucial for regaining strength and adapting to these changes, allowing you to return to many daily activities with some modifications**.	After scoliosis surgery, particularly spinal fusion, your range of motion in the spine may be reduced, especially in bending and twisting movements. The extent of this limitation depends on the location and amount of spine fused**. Post-surgery rehabilitation, including physical therapy, is crucial for adapting to these changes and regaining strength and flexibility.**	
Median rating = 2.0<br>STD = 0.94						**0.0**
It's essential to consult with a medical professional, especially an orthopedic surgeon or a specialist in spine disorders, to get personalized advice and answers based on individual circumstances.						
